# Mapping the multicausality of Alzheimer’s disease through group model building

**DOI:** 10.1007/s11357-020-00228-7

**Published:** 2020-08-11

**Authors:** Jeroen F. Uleman, René J. F. Melis, Rick Quax, Eddy A. van der Zee, Dick Thijssen, Martin Dresler, Ondine van de Rest, Isabelle F. van der Velpen, Hieab H. H. Adams, Ben Schmand, Inge M. C. M. de Kok, Jeroen de Bresser, Edo Richard, Marcel Verbeek, Alfons G. Hoekstra, Etiënne A. J. A. Rouwette, Marcel G. M. Olde Rikkert

**Affiliations:** 1 Department of Geriatric Medicine, Radboudumc Alzheimer Center, Donders Institute for Brain, Cognition and Behaviour, Radboud University Medical Center, Reinier Postlaan 4, 6525GC Nijmegen, The Netherlands; 2 Institute for Advanced Study, Amsterdam, The Netherlands; 3 Computational Science Lab, Faculty of Science, University of Amsterdam, Amsterdam, The Netherlands; 4 Institute for Management Research, Radboud University, Nijmegen, The Netherlands; 5 Department of Geriatric Medicine, Radboud Institute for Health Sciences, Radboud University Medical Center, Nijmegen, The Netherlands; 6 Molecular Neurobiology, Groningen Institute for Evolutionary Life Sciences (GELIFES), University of Groningen, Groningen, The Netherlands; 7 Department of Physiology, Radboud Institute for Health Sciences, Radboud University Medical Center, Nijmegen, The Netherlands; 8 Liverpool John Moores University, Liverpool, United Kingdom; 9 Donders Institute for Brain, Cognition and Behaviour, Radboud University Medical Center, Nijmegen, The Netherlands; 10 Division of Human Nutrition and Health, Wageningen University, Research, Wageningen, The Netherlands; 11 Department of Epidemiology, Department of Radiology and Nuclear Medicine, Erasmus MC, University Medical Center Rotterdam, Rotterdam, The Netherlands; 12 Department of Psychology, University of Amsterdam, Amsterdam, The Netherlands; 13 Department of Public Health, Erasmus MC, University Medical Center Rotterdam, Rotterdam, The Netherlands; 14 Department of Radiology, Leiden University Medical Center, Leiden, The Netherlands; 15 Department of Neurology, Donders Institute for Brain, Cognition and Behaviour, Radboud University Medical Centre, Nijmegen, The Netherlands; 16 Departments of Neurology and Laboratory Medicine, Donders Institute for Brain, Cognition and Behaviour, Radboud University Medical Centre, Nijmegen, The Netherlands; 17 Department of Clinical Genetics, Erasmus MC, University Medical Center Rotterdam, Rotterdam, The Netherlands; 18 Department of Radiology and Nuclear Medicine, Erasmus MC, University Medical Center Rotterdam, Rotterdam, The Netherlands

**Keywords:** Systems thinking, Alzheimer’s disease, Causal loop diagram, Multicausal, Complexity, Group model building, Centrality

## Abstract

Alzheimer’s disease (AD) is a complex, multicausal disorder involving several spatiotemporal scales and scientific domains. While many studies focus on specific parts of this system, the complexity of AD is rarely studied as a whole. In this work, we apply systems thinking to map out known causal mechanisms and risk factors ranging from intracellular to psychosocial scales in sporadic AD. We report on the first systemic causal loop diagram (CLD) for AD, which is the result of an interdisciplinary group model building (GMB) process. The GMB was based on the input of experts from multiple domains and all proposed mechanisms were supported by scientific literature. The CLD elucidates interaction and feedback mechanisms that contribute to cognitive decline from midlife onward as described by the experts. As an immediate outcome, we observed several non-trivial reinforcing feedback loops involving factors at multiple spatial scales, which are rarely considered within the same theoretical framework. We also observed high centrality for modifiable risk factors such as social relationships and physical activity, which suggests they may be promising leverage points for interventions. This illustrates how a CLD from an interdisciplinary GMB process may lead to novel insights into complex disorders. Furthermore, the CLD is the first step in the development of a computational model for simulating the effects of risk factors on AD.

**Electronic supplementary material:**

The online version of this article (10.1007/s11357-020-00228-7) contains supplementary material, which is available to authorized users.

## Introduction

### Alzheimer’s disease is multicausal

Alzheimer’s disease (AD) is a complex disorder with a multicausal etiology that remains difficult to elucidate despite the many and diverse research efforts, which focus on various causal mechanisms. For instance, the most prominent causal hypothesis concerns the aggregation of amyloid-beta protein in the brain, leading to the formation of senile plaques and neuronal dysfunction (Hardy and Selkoe 2002; Karran et al. 2011). However, the relationship between this amyloid cascade and the onset and progression of AD lacks specificity (Mortimer 2012) and weakens with increasing age and frailty (Savva GM et al. 2009; Wallace et al. 2019). Furthermore, clinical trials based on this hypothesis have not yet yielded effective disease-modifying treatments (Cummings et al. 2019; Karran et al. 2011). One may thus tentatively conclude that while the amyloid cascade appears to play a role in AD, it is far from the complete story (Pimplikar 2009).

Indeed, in recent years, researchers argue that unraveling this complexity at the systemic level may be crucial for the development of efficacious treatments (Pomorska and Ockene 2017; Tang et al. 2019; Rollo et al. 2016). This is supported by many additional mechanisms that are probably involved in the etiology of AD, such as glucose metabolism and oxidative stress (Butterfield and Halliwell 2019; Nunomura et al. 2006), vascular dysfunction (Sweeney et al. 2019), and inflammation (Newcombe et al. 2018; Heneka et al. 2015). This multicausality is increasingly recognized (Fotuhi et al. 2009; Sweeney et al. 2019), but a comprehensive understanding of the interactions between these causes is lacking. Such an overarching understanding of AD requires intense interdisciplinary collaboration between various traditional scientific disciplines that now largely focus on a single scale or mechanism (Kuljis 2009; van Dijk et al. 2015), supported by methodologies that are largely novel in this field, originating from complexity science and the study of complex adaptive systems (Braithwaite et al. 2018).

### Applying systems thinking to Alzheimer’s disease

Systems thinking offers a methodology to realize such a holistic, interdisciplinary approach. In this way, it helps to understand the behavior of complex adaptive systems such as organs and organisms, and may thus also be useful in disentangling the interaction and feedback mechanisms that lead to cognitive decline. It offers validated methods for identifying causal mechanisms and the interactions between them. Unsurprisingly, systems thinking is being increasingly recognized and utilized in biomedical and neuroscience literature (Wittenborn et al. 2016; Kenzie et al. 2018; Vandenbroeck et al. 2007).

An important concept in systems thinking is the causal loop diagram (CLD), which is a conceptual model of relevant mechanisms and their interactions. In this paper, we report on the first systemic CLD for AD, which was realized using group model building (GMB). The CLD comprises the combined conceptual model of an extensive interdisciplinary group of AD researchers and computational modelers, checked against the scientific literature. We do not present the CLD as a complete representation of reality but rather as a summary of knowledge, agreed upon by this specific group of experts, which helps elucidate central processes involved in the onset and progression of AD.

## Methods

### Causal loop diagram

CLDs visualize the known or assumed causal structure of a system and consist of variables and connections drawn as arrows between the variables. These connections have specific directions, in line with the underlying causal relationships, and typically have positive or negative polarity (Bala et al. 2017). A positive connection (+) implies that when the causal variable changes in one direction, the variable it connects to changes in the same direction; a negative connection (-) implies that when the causal variable changes in one direction, the variable it connects to changes in the opposite direction. A direct connection between two variables implies an effect that does not go via any of the other variables in the CLD.

As the name suggests, an important feature of CLDs is the presence of feedback loops. Feedback mechanisms are important drivers of the nonlinear behavior of dynamical systems (Forrester 2009; Littlejohns et al. 2018) and can reinforce or balance the impact of stimuli. Reinforcing loops have a self-strengthening effect and can push the system out of balance, whereas balancing loops have a self-limiting effect and promote equilibrium restoration and thus support homeostasis. Identifying reinforcing loops may be particularly important for understanding the amplification of inter-individual differences in risk profiles, which can result in substantial heterogeneity (Sterman 2000) in terms of onset and progression of AD.

Feedback loops with only two variables are “direct,” while loops consisting of more than two variables are defined as “indirect.” When the variables in a loop all occur in the same characteristic spatial scale (e.g., cellular), the loop is referred to as “within-scale.” When the variables in the loop occur in different spatial scales, the loop is referred to as “cross-scale.” Identifying cross-scale loops is of particular interest because they can be easily overlooked when studying the system exclusively from one spatial scale or scientific domain. From a systems perspective, all known and hypothesized causal relationships between variables should be taken into account, even those that are separated by spatial or temporal scales (Forrester 1971).

### Group model building

Group model building (GMB) is a participatory method for involving experts in developing conceptual or computational models (Vennix 1999; Andersen et al. 2007; Hovmand et al. 2012). In GMB, the mental models and assumptions of experts are elicited and captured in a shared model, which is the result of consensus in the group (Bérard 2010; Vennix 1996). The resulting model is a summary of explicit, tested, and integrated knowledge of the group.

We organized two GMB sessions with two weeks in between, specifically aimed at developing a CLD. We aimed to include a sufficiently large variety of expertise while ensuring some overlap. To this end, we formulated an expertise table (see Supplementary Materials). The group consisted of thirteen domain experts from a wide scope of AD research and two experts in complexity research and computational modeling. The expressed purpose of our GMB sessions was to structurally explain the difference between cognitive decline trajectories in sporadic AD compared with normal aging, starting from midlife. We defined AD using clinical criteria (McKhann et al. 2011) and focused on sporadic AD, meaning that we did not take into account the genes associated with familial AD: PSEN1, PSEN2, and APP (Dorszewska et al. 2016).

During the GMB sessions, a facilitator (EAJAR) guided the group discussions and a computational modeler (JFU) sketched resulting versions of the CLD on a screen using Vensim PLE (Version 7.3.5) (Vensim 2019). We alternated between phases of divergence and convergence. During divergence, the nominal group technique (Gustafson et al. 1986) was applied, during which the experts individually wrote down variables to add to the CLD. These variables were then collected and displayed on the screen for all participants to see. During convergence, debate was encouraged between experts to reach a consensus about the addition or deletion of certain variables or connections. Any proposed additions were discussed in the group and only added to the CLD when objections were resolved and a consensus was reached. During the second session, three subgroups were formed based on the main research topics of the experts (see the “Results” section). The subgroups were tasked with checking the parts of the model most closely connected to their area of expertise. The changes made by these subgroups were presented and discussed with the rest of the group.

After each GMB session, a summary report containing all results, considerations, and discussion points was sent to the participating experts. The experts were then asked to provide feedback on the reports of both sessions. After the final session, the experts provided scientific evidence in the form of literature references for each of the connections in the CLD together with the modeling team. This resulted in several additional changes to the CLD, which were communicated back to and accepted by the group.

### Network analysis

The causal connections in the resulting CLD can be interpreted to form a network structure. For network processes, a typical initial analysis is to identify structurally important, or “central”, variables. Such an analysis can also help pinpoint central drivers of disease in CLDs (McGlashan et al. 2016). To this end, we calculated the betweenness centrality (BC) and closeness centrality (CC) for each of the variables in the CLD.

In a CLD, shortest paths exist between each pair of variables, which correspond to sequences of connections that contain the least number of mediating variables in the causal pathway. If mediating variables are seen as possible points of interfering with, or adding noise to, the causal pathway then it follows that shortest paths tend to be the strongest causal pathways. As such, measures that are defined in terms of shortest paths, such as BC and CC (Brandes et al. 2016), could be informative of the importance of factors in a causal way. In particular, variables with high BC lie on many of the shortest paths between other variables, making them potentially important connectors (Ahmed 2017). That is, mediating multiple different causal pathways simultaneously. These variables could be succinct points for diagnosing aberrant system dynamics and also serve as potential targets for interventions. Complementarily, variables with high closeness have shortest paths to many other variables in the CLD with a short distance, i.e., few mediating variables, rendering them potentially efficient spreaders of information (Ahmed 2017). These variables could thus also be good starting points for interventions.

The normalized BC of variable *v* is calculated using Eq. 1, where *N* is the number of variables in the CLD, *σ*(*s*, *t*) is the number of shortest paths between variables *s* and *t*, and *σ*(*s*, *t*|*v*) is the number of those shortest paths that pass through variable *v*.

1 $$ \text{BC}_{v} = \frac{1}{(N-1)(N-2)} \sum\limits_{s, t} \frac{\sigma(s,t|v)}{\sigma(s,t)}  $$

The CC of variable *v* is calculated using Eq. 2, where *n* − 1 is the number of reachable variables and *d*(*v*, *u*) is the distance of the shortest path from variable *v* to variable *u*.

2 $$ \text{CC}_{v} = (n-1) \frac{1}{{\sum}^{n-1}_{u=1} d(v,u)}  $$

In order to test the robustness of these measures to possible errors in the structure of the CLD, we created 1000 alternative CLDs, each with 5 mutations in them compared with the original. These mutations were equiprobable and consisted of either a random rewiring (e.g., some connection A->B is replaced by connection A->C) or the random addition or deletion of a connection. Centrality measures were calculated for each of these alternative CLDs and the interquartile ranges of the resulting distributions were used to construct error bars.

Another way of assessing the behavior of the system represented by a CLD is to study its feedback loops, which can result in nonlinear influences of the variables (Forrester 2009). Important within- and cross-scale feedback loops are therefore visualized in the CLD and the potential relevance of several examples to AD will be discussed.

## Results

**Fig. 1:**
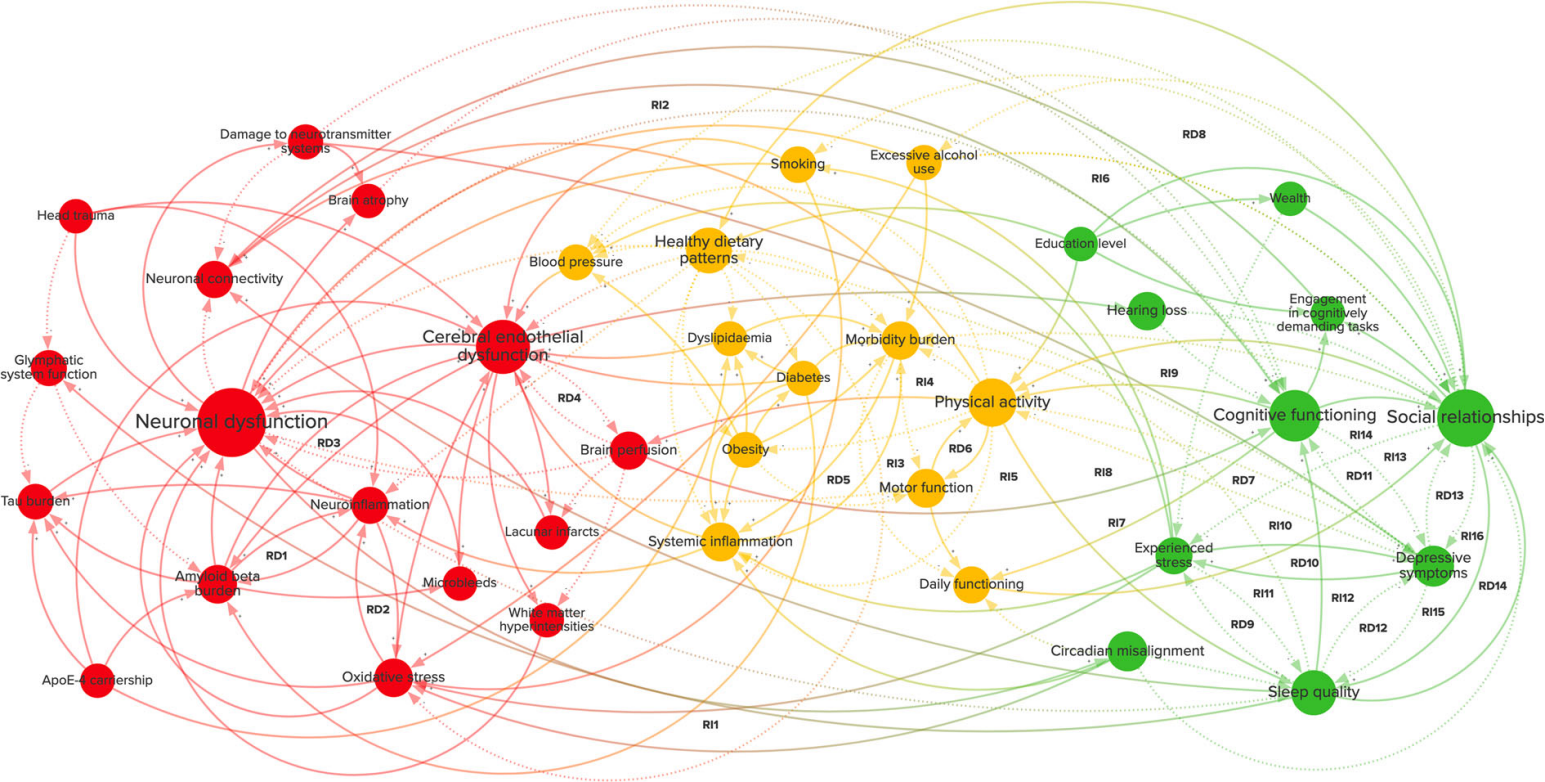
Causal loop diagram of sporadic Alzheimer’s disease. The diagram contains related variables and causal connections between them. The diagram is divided into variables related to brain health (red), physical health (yellow), and psychosocial health (green). A positive connection (+, solid line) represents an effect in the same direction, whereas a negative connection (-, dotted line) represents an effect in the opposite direction. The size of the variables is scaled by their betweenness centrality. RD1–14 and RI1–16 represent direct and indirect reinforcing feedback loops

The CLD shown in Fig. 1 is a graphical depiction of the combined multidisciplinary knowledge of fifteen experts. These experts proposed, discussed, and agreed on 38 variables and 150 connections between them. The variables in the CLD were divided into three categories: brain health, physical health, and psychosocial health. These categories correspond to the subgroups in which the experts were divided and can be found in the supplementary material, together with definitions of each of the variables as well as supporting evidence for the connections. An interactive visualization of the CLD was created in Kumu (2019) which can be found https://cldforad.kumu.io/mapping-the-complex-multicausality-in-alzheimers-disease?token=l6CvrnWTeDcLkHRl.

### Variable centrality

**Fig. 2:**
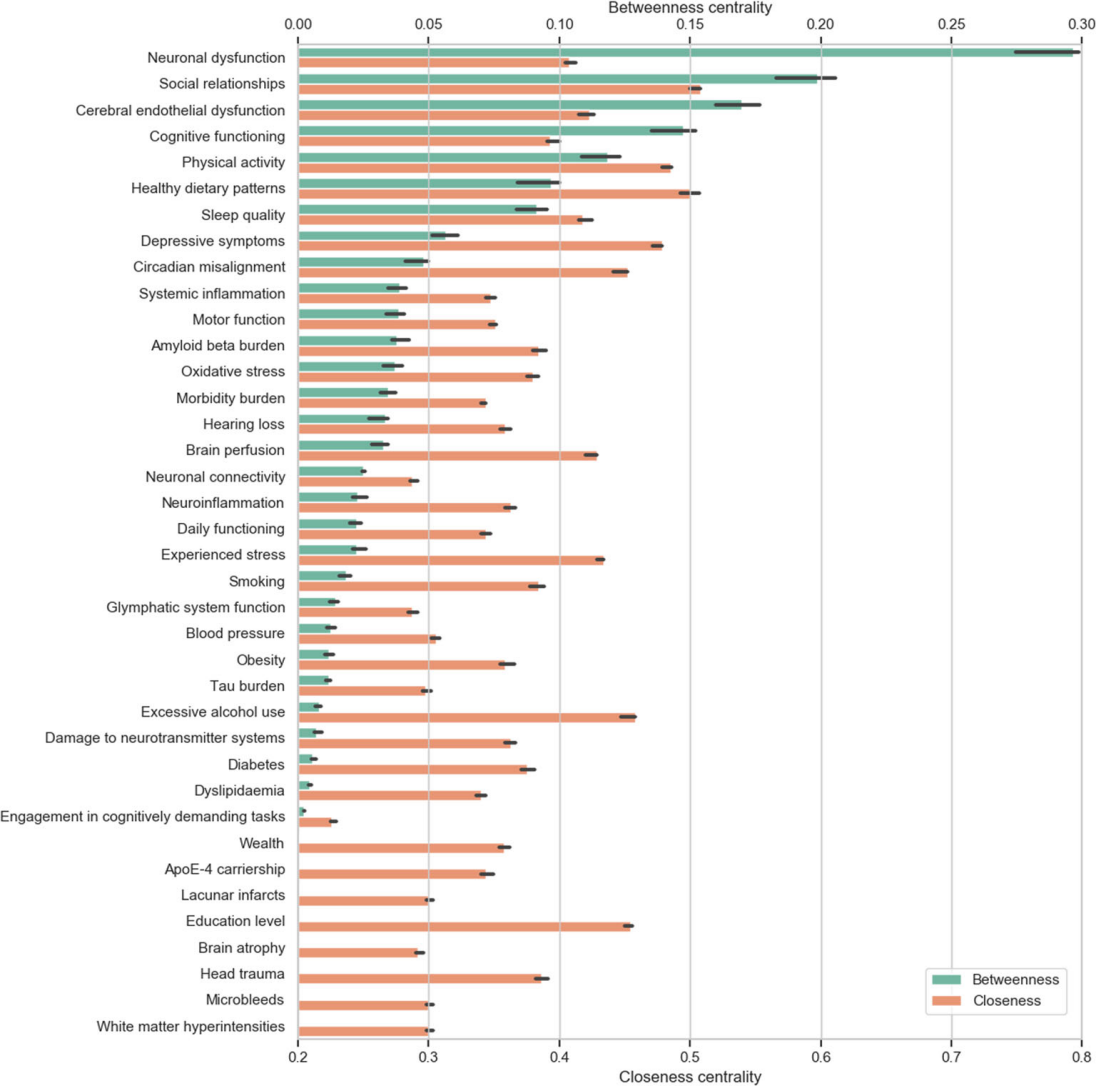
The betweenness and closeness centralities of the variables in the causal loop diagram (Fig. 1). The error bars represent the interquartile range of 1000 mutated diagrams with each 5 random rewirings, additions of deletions

The centrality measures for each of the variables in the CLD are given in Fig. 2. Spearman’s rank-correlation coefficient between the two measures (BC and CC) is *ρ* = 0.50. Neuronal dysfunction has a very high BC and is clearly an important variable in the CLD, connecting many pathophysiological variables to structural brain damage, daily and cognitive functioning. The central position of neuronal dysfunction (and neuronal loss, which is the ultimate state of neuronal dysfunction) in the CLD may indicate its role as a final common pathway that is implicated in AD: at the level of the brain most mechanisms cause cognitive decline via neuronal dysfunction. Furthermore, at the interface of the circulation and brain, cerebral endothelial dysfunction appears to have an important role connecting risk factors such as physical inactivity, obesity, dyslipidemia, diabetes, and hypertension to brain pathology. That being said, the experts also agreed on paths to neuronal dysfunction that do not go via cerebral endothelial dysfunction, such as effects of smoking and alcohol use via DNA methylation (Corley et al. 2019; Xu et al. 2019). Lifestyle factors like social relationships, physical activity, healthy dietary patterns, sleep quality, and depressive symptoms have high BC as well as CC, indicating that they may also be important drivers of vascular and neuronal dysfunction. Furthermore, sleep deprivation and depressive symptoms can exert direct effects that result in cognitive impairment (Alhola and Polo-Kantola 2007; Rock et al. 2014).

### Exogenous variables

Education level, head trauma, and apoE-4 carriership are exogenous to the CLD and thus have a BC of 0. The CC of these variables, however, is relatively high, especially of education level, suggesting that it is an important contributor to cognitive decline (Wilson et al. 2009). Exogenous variables influence the system without being influenced *by* it. They can be seen as part of the context of an individual and their history. They can also be seen as entry points into the system. ApoE-4 carriership might, for example, play a role in dyslipidemia (Marais 2019) and could thereby contribute to AD pathogenesis.

### Within and cross-scale feedback loops

Several feedback loops have been identified in the CLD and are annotated in Fig. 1. An overview of the feedback loops is also given in Table 1. All the direct reinforcing loops have been denoted as RD1–RD14. Similarly, indirect reinforcing loops, limited to three variables, have been denoted as RI1–RI6. None of these loops are balancing, suggesting that balancing loops may be of less importance than reinforcing loops for understanding cognitive decline trajectories in sporadic AD.

**Table 1:** Feedback loops in the causal loop diagram (Fig. 1)

Loop	1st variable	2nd variable	3rd variable
RD1	Amyloid beta burden	Neuroinflammation	-
RD2	Oxidative stress	Neuroinflammation	-
RD3	Amyloid beta burden	Cerebral endothelial dysfunction	-
RD4	Brain perfusion	Cerebral endothelial dysfunction	-
RD5	Systemic inflammation	Morbidity burden	-
RD6	Motor function	Physical activity	-
RD7	Physical activity	Depressive symptoms	-
RD8	Excessive alcohol use	Social relationships	-
RD9	Sleep quality	Experienced stress	-
RD10	Depressive symptoms	Experienced stress	-
RD11	Depressive symptoms	Cognitive functioning	-
RD12	Depressive symptoms	Sleep quality	-
RD13	Depressive symptoms	Social relationships	-
RD14	Sleep quality	Social relationships	-
RI1	Oxidative stress	Neuronal dysfunction	Circadian misalignment
RI2	Physical activity	Neuronal connectivity	Cognitive functioning
RI3	Obesity	Motor function	Physical activity
RI4	Morbidity burden	Motor function	Physical activity
RI5	Brain perfusion	Physical activity	Cognitive functioning
RI6	Neuronal connectivity	Cognitive functioning	Engagement in cognitively demanding tasks
RI7	Morbidity burden	Daily functioning	Social relationships
RI8	Physical activity	Sleep quality	Cognitive functioning
RI9	Physical activity	Depressive symptoms	Social relationships
RI10	Physical activity	Sleep quality	Social relationships
RI11	Experienced stress	Depressive symptoms	Sleep quality
RI12	Experienced stress	Sleep quality	Depressive symptoms
RI13	Experienced stress	Depressive symptoms	Social relationships
RI14	Social relationships	Depressive symptoms	Cognitive functioning
RI15	Depressive symptoms	Sleep quality	Social relationships
RI16	Depressive symptoms	Social relationships	Sleep quality

Direct reinforcing loops (RD) consist of two variables and the indirect reinforcing loops (RI) consist of three variables

Within-scale loops can significantly impact local pathological processes and may fall within the scope of one scientific domain. For example, at the level of the brain, loop RD1 may aggravate neuronal dysfunction as aggregates of amyloid-beta protein may activate microglia, leading to increased release of TNF-*α*, which can inhibit the phagocytosis of amyloid-beta (Tejera and Heneka 2016).

An example of a cross-scale loop is RI1. Oxidative stress can lead to an impairment of glucose metabolism and thereby to neuronal dysfunction (Butterfield and Halliwell 2019). This could lead to circadian misalignment when it includes damage to melanopsin-expressing retinal ganglion cells (Feng et al. 2016) and could thereby result in exacerbated oxidative stress (Musiek and Holtzman 2016).

**Fig. 3:**
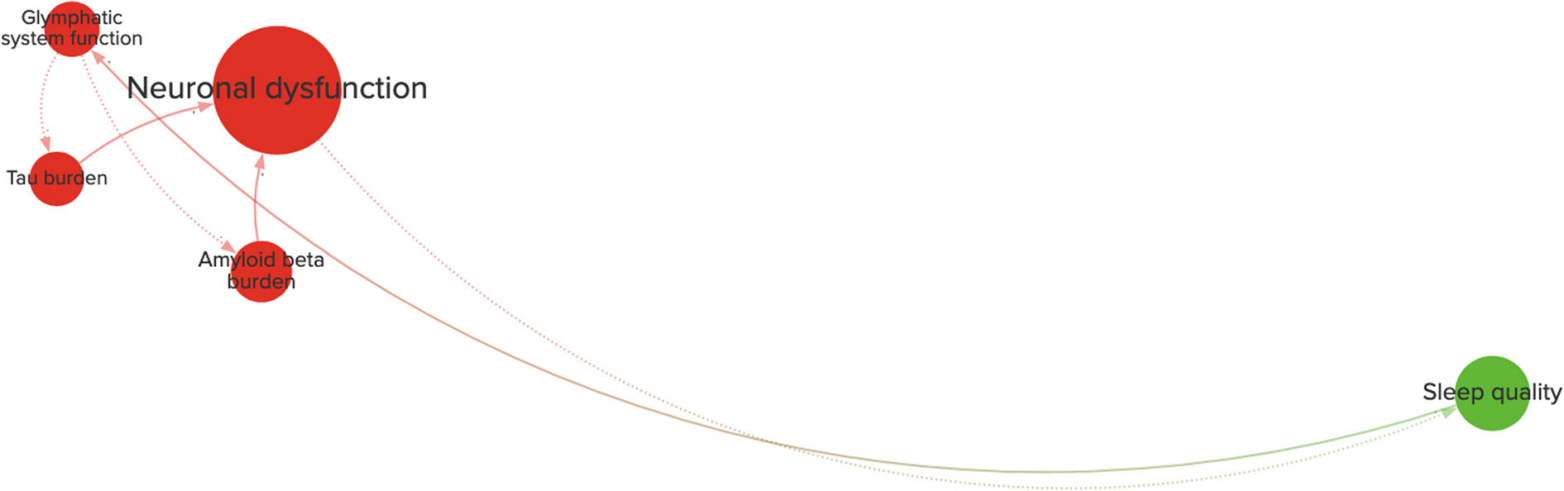
An example of a reinforcing cross-scale feedback loop with four variables (from Fig. 1). The diagram is divided into variables related to brain health (red) and psychosocial health (green). A positive connection (+, solid line) represents an effect in the same direction, whereas a negative connection (-, dotted line) represents an effect in the opposite direction

Loops with more than three variables also exist and, although they are sometimes difficult to recognize, they may have important effects on the system (Kenzie et al. 2018). These loops can often exist between variables at multiple spatial or temporal scales and may be related to various scientific domains. An example of a longer cross-scale loop is shown in Fig. 3. A disturbance of sleep quality can lead to an impairment of glymphatic system functioning (Rasmussen et al. 2018). This may aggravate amyloid-beta as well as tau pathology (Rasmussen et al. 2018), potentially leading to neuronal dysfunction and ensuing reductions in sleep quality (Wang and Holtzman 2020).

### Example: Physical activity

**Fig. 4:**
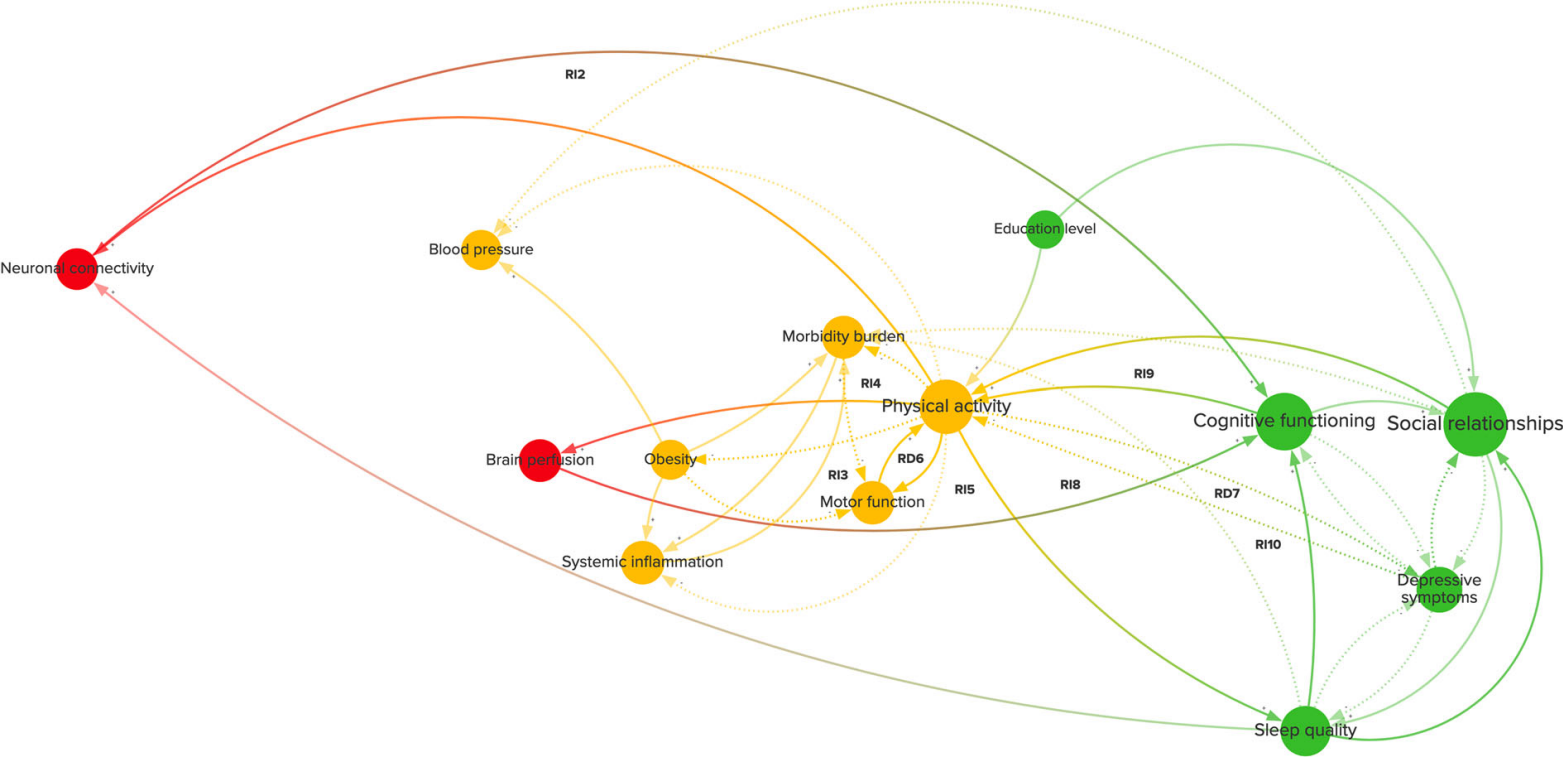
Feedback loops (from Fig. 1) that include physical activity. This diagram shows the variables, causal connections, and reinforcing direct (RD6–7) and indirect (RI2–5, RI8–10) feedback loops related to physical activity. The diagram is divided into variables related to brain health (red), physical health (yellow), and psychosocial health (green). A positive connection (+, solid line) represents an effect in the same direction, whereas a negative connection (-, dotted line) represents an effect in the opposite direction

Physical activity is a good example of how risk factors can exert their influence on the system through the feedback loops they are involved in. Physical activity is a well-established modifiable risk factor (Livingston et al. 2017) and has high centrality in the CLD. In Fig. 4, physical activity is highlighted in the CLD with each connection, variable, and feedback loop that it is connected to. Physical activity is part of seven indirect and two direct reinforcing feedback loops, one with motor function (RD6) and one with depressive symptoms (RD7).

Increasing physical activity may reduce the onset of multiple morbidities (e.g., diabetes and obesity) by improving cardiovascular and metabolic health (Gallaway et al. 2017) and, in turn, reduce the occurrence of multimorbidity, all of which may prevent corresponding reductions in motor function (Calderón-Larrañaga et al. 2019) and thereby result in more physical activity through RI4 as well as RD6. Increasing physical activity might also reduce depressive symptoms by increasing brain-derived neurotrophic factor levels (RD7) (Mandolesi et al. 2018). These reductions in depressive symptoms could also lead to improved social relationships (VanderWeele et al. 2012), which can further stimulate physical activity (Barth et al. 2010) (RI9). Loops like RD7, which are nested in longer loops, could induce further unexpected nonlinear effects on the system.

Physical activity is also part of two cross-scale loops that involve cognitive functioning. Increasing physical activity may have beneficial effects on brain perfusion (RI5) (Mandolesi et al. 2018) as well as neuronal connectivity (RI2) via FNDC5/irisin improving synaptic plasticity (Lourenco et al. 2019), both of which can have beneficial effects on cognitive functioning (Ogoh 2017; Koen and Rugg 2019). Cross-scale loops often include variables that are not only separated in space but often also in time. For example, it could take many years before a gradual loss of neuronal connectivity results in a notable loss of cognitive functioning (Koen and Rugg 2019). Loops like RI2 could, nevertheless, significantly influence AD onset and progression over such time scales.

**Fig. 5:**
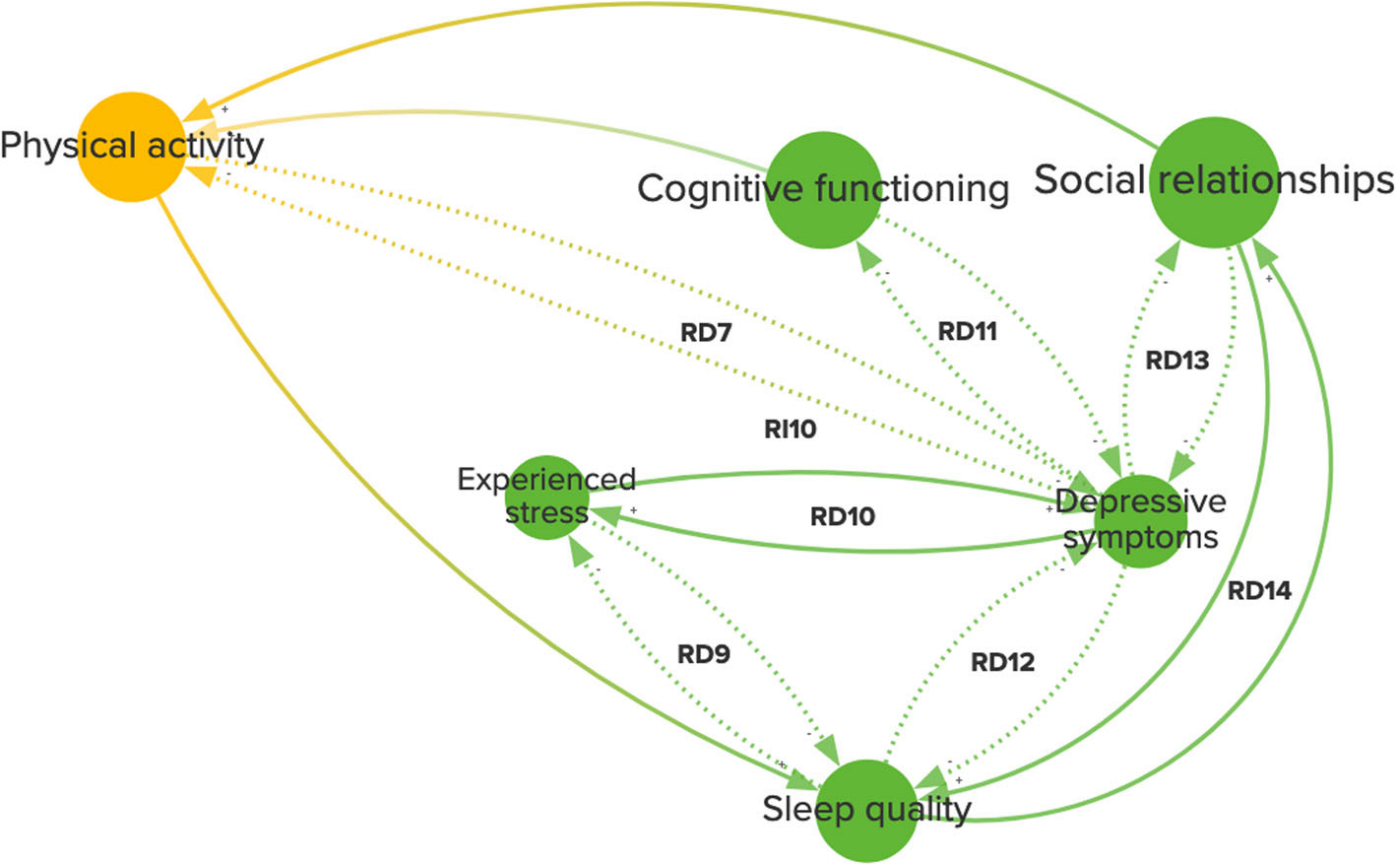
A cluster of long and nested feedback loops in the causal loop diagram (Fig. 1). This diagram shows variables, causal connections, and reinforcing feedback loops related to physical health (yellow) and psychosocial health (green). A positive connection (+, solid line) represents an effect in the same direction, whereas a negative connection (-, dotted line) represents an effect in the opposite direction

Besides its bidirectional relationship with depressive symptoms (R7), physical activity is also closely related to other potentially modifiable factors with high centrality, specifically social relationships, and sleep quality (RI10). Figure 5 shows that depressive symptoms are involved in many feedback loops, such as with cognitive functioning (RD11), sleep quality (RD12), experienced stress (RD10), and social relationships (RD13), which also have loops amongst each other (RD9, RD14). This strong interconnection is also apparent in several indirect loops (RI15–16, RI11–12), in which these direct loops are nested, and even longer overlapping loops, such as between physical activity, sleep quality, social relationships, and depressive symptoms (Fig. 5). Such longer, cross-scale loops, containing variables with high centrality and having several loops nested within them, may be crucial for understanding the onset of AD as well as the influence of potentially modifiable risk factors.

## Discussion

CLDs can be used as a system-wide map of the multicausality of risk factors and pathophysiology of complex biomedical issues (Wittenborn et al. 2016; Kenzie et al. 2018). We have utilized this tool to summarize expert knowledge on the multicausal etiology of sporadic AD across scientific disciplines. The CLD contains variables and causal relationships formulated in a manner that this interdisciplinary group of experts agreed on. This paves the way for intensified interdisciplinary collaboration within the AD field and shows that GMB can be utilized for eliciting, contrasting, and integrating knowledge from AD experts across various scientific domains.

Although its qualitative nature makes it difficult to draw definite conclusions, the current CLD may already have several implications. The BC and CC point at a significant role for modifiable risk factors in AD. High centrality makes overall effects on cognitive decline via such factors more likely to occur and also underscores that such factors are endogenous to the disorder and bidirectionally related to underlying pathophysiological processes. This supports the idea that multi-domain lifestyle interventions may be promising, particularly when intervening on social relationships, physical activity, diet, sleep, and depression. Their high centrality suggests that they mediate and impact on numerous other mechanisms relevant to AD. This is supported by results from the 2-year Finnish Geriatric Intervention Study to Prevent Cognitive Impairment and Disability (FINGER) study, which suggests that multi-domain interventions may maintain cognitive functioning in at-risk elderly people (Ngandu et al. 2015). Furthermore, the relatively high BC of cerebral endothelial dysfunction supports the increased recognition of the role of cerebrovascular dysfunction in AD and the impact of systemic vascular health on brain health (Sweeney et al. 2019).

At the very least, the CLD emphasizes the necessity of widely recognizing AD as a complex and multicausal condition. The many relations between the multitude of variables in the system could lead to unexpected results when intervening in them. It appears unlikely that a few variables or a single feedback loop can be isolated from the rest of the system and fully understood independently. For instance, potentially beneficial effects of an intervention may be compensated by adverse effects materializing along another pathway. It also demonstrates the need to develop such CLDs: it is highly unlikely that an individual domain expert has an equally encompassing mental map as the resulting systemic CLD. The multicausality as illustrated by the CLD poses challenges to analysis methods that are purely based on statistical associations, such as structural equation models (Shen et al. 2020). It reveals the need for a causal structural understanding of AD as a system of intersecting causal pathways and loops, suggesting that there may not even be a single, localized intervention that can counter the disease progression by itself.

Importantly, the CLD approach to knowledge synthesis we utilized here is the basis for a computational implementation in the form of a system dynamics model. As a next step of the empirical cycle (Hoekstra et al. 2019), it could be used to simulate a variety of intervention scenarios on modifiable risk factors over time, which may then be tested experimentally. Rather than going immediately from theory and knowledge integration to experimental validation, simulation modeling should allow for a better understanding of the mechanisms underlying intervention scenarios and could thereby help identify potential leverage points in the system. This increased understanding may increase the prior probability of such scenarios and limit the number of failed empirical intervention studies (Ioannidis 2005, 2016). Given the large number of unsuccessful clinical trials (Cummings et al. 2019), this may be very important indeed.

The presence of cross-scale feedback loops further indicates a need for sustained interdisciplinary collaborations that consider long-range interactions between variables typically associated with different scientific disciplines. Many cross-scale feedback loops that can be found in Fig. 1 are longer than three variables, such as the loop in Fig. 5. Longer loops are not considered in Table 1 because they are influenced by so many variables that their interpretation becomes ambiguous without quantitative information. Such loops could nevertheless turn out to be critical to the behavior of the system in a quantitative analysis.

Despite our systematic application of GMB, there is subjectivity involved in the process. The CLD is to some extent unique to this specific group of experts and may not be easily replicated in a different group. However, the ordering of the centrality measures of the variables was robust as evidenced by the error bars in Fig. 2, which rarely overlapped. This suggests that small errors made in the wiring of the diagram would not have resulted in different qualitative conclusions, especially for the variables with high centrality. Furthermore, due to the wide range of expertise included in our group and the embedding of all connections in the scientific literature, such errors are likely minimized. To assess the influence of bias in the variable selection on the centrality measures, we used cerebrovascular pathology as an example and tested whether omitting white matter hyperintensities, microbleeds, and lacunar infarcts from the CLD would alter the BC ranking of cerebral endothelial dysfunction. This test yielded minimal changes and only slightly lowered the BC of endothelial dysfunction to a comparable level as cognitive functioning. Limiting the level of detail was a deliberate choice by the group and resulted in a more comprehensible CLD. We aimed to include the most important mechanisms that contribute to AD rather than to be completely exhaustive. Consequently, several processes have been combined into aggregate variables. For example, cellular processes like mitochondrial dysfunction and endoplasmic reticulum stress have been aggregated in overarching terms such as neuronal dysfunction. Questions at higher levels of detail can thus not yet be addressed using the current CLD. Future efforts could extend the CLD by increasing its level of detail, particularly at the (sub-)cellular scale.

A pitfall of CLDs is that they exclusively contain qualitative information. Accordingly, the centrality measures of the variables might change considerably when taking into account the strength of the connections. The use of centrality measures for causal importance has also been disputed recently (van Elteren and Quax 2019; Dablander and Hinne 2019) since it does not take into account the specific dynamics represented by the variables and connections. That being said, high centrality variables were found to correspond to known drivers of childhood obesity in a CLD (McGlashan et al. 2016). Therefore, as a first approximation and while dynamics are not yet modeled quantitatively, variables with high centrality might—on the whole—be responsible for a significant proportion of the effect on cognitive decline in our CLD as well. At this point, a network-structural approach for proposing causally relevant variables is the best we can do. The moderate-to-large monotonic correlation between BC and CC further suggests that, although they overlap, these measures also have complementary value. For example, CC was useful for comparing the exogenous variables.

The CLD remains a reflection of the knowledge of our group of experts and of the available scientific evidence. Hence, some connections that exist in reality may have been omitted in the CLD. The implications of this uncertainty will be assessed using uncertainty quantification (UQ) techniques applied to the system dynamics model. Because our CLD is the result of GMB and our review of scientific literature was not fully systematic, the CLD by itself does not yet provide insight into which of the connections require further investigation. However, when computationally implemented, the CLD could become a valuable, complementary tool to systematic reviews for identifying potentially interesting empirical research targets. In the sequel to this work, UQ and sensitivity analysis techniques applied to the system dynamics model will be used to identify which of the connections are important for model predictions and which would require additional data in order to be estimated with high confidence. Through these means, we will investigate which parts of the model require further scientific research. The lack of balancing feedback loops is likely a consequence of our aim of mapping the mechanisms leading to cognitive decline in AD, rather than homeostatic and resilience mechanisms. For a fuller picture, future efforts could also aim to chart out balancing loops to give a more complete representation of the system that is less skewed to factors that promote the development and progression of clinically manifest AD and instead also includes factors that counteract mechanisms of disease progression.

Although the CLD is, just like any other model (Sterman 2002), an incomplete representation of reality, it can still be used for educational purposes, hypothesis generation, and the identification of confounder and collider variables to consider in statistical analyses (VanderWeele 2019). Furthermore, the CLD is the first step in an iterative process of not only developing computational models but also further mapping out the processes implicated in AD. As such, it could become increasingly exhaustive, reaching levels of detail like the Foresight system map developed for obesity (Vandenbroeck et al. 2007).

We have the ambition of turning this work into an ongoing effort of the scientific community to expand and parameterize the model we developed, but a rigorous methodology for such a community-level approach must still be developed. For now, we encourage AD researchers to use our online version of the CLD to make suggestions for improvements or extensions using the designated comment function. Additional systematic methods for triangulation and extending the CLD might entail word cloud analyses (Atenstaedt 2012) in order to identify important variables that are not currently present in the diagram, or systemic reviews which could be conducted for every connection that might plausibly exist between any of the variables in the CLD. In addition, quality assessment criteria could be developed for the application of GMB to complex scientific issues, similar to the PRISMA criteria for systematic reviews (Moher et al. 2009). This would promote the quality and wide-scale reliable application of GMB and of mixed quantitative and qualitative methods.

## Conclusion

This work demonstrates the relevance and feasibility of using systems thinking and GMB to improve our understanding of the biopsychosocial causality in AD. Our study specifically serves as a proof-of-concept of this innovative method in the AD field. The general notion that AD is complex and multicausal is supported by the large number of variables, causal connections, and feedback loops that we have identified in the system, which exist at multiple spatial and time scales. As such, this work is complementary to scientific approaches confined to a single mechanism that cannot fully account for the interactive behavior of the network of multiple causes at different scales.

We will use the CLD for the development of a system dynamics model. This model will be calibrated and validated using empirical data of longitudinal cohort studies and randomized clinical trials. By these means, it may become an important tool for simulating and predicting cognitive and global functional performance effects of multi-domain lifestyle or drug interventions. We believe that GMB and systems thinking should be further developed for and applied to complex multicausal conditions, particularly AD.

## Electronic supplementary material

Below is the link to the electronic supplementary material.

(XLSX 48.5 KB)
